# COVID-19: Obesity, deprivation and death

**DOI:** 10.7189/jogh.10.020389

**Published:** 2020-12

**Authors:** A Hamish RW Simpson, Cameron J Simpson, Helen Frost, Susan C Welburn

**Affiliations:** 1 Edinburgh Medical School, University of Edinburgh, Edinburgh, Scotland; 2 NHS Scotland, Edinburgh, Scotland; 3 Edinburgh Napier University, Edinburgh, Scotland; 4 Infection Medicine, College of Medicine and Veterinary Medicine, University of Edinburgh, Edinburgh, Scotland; 5 Zhejiang University-University of Edinburgh Institute, Zhejiang University School of Medicine, Zhejiang, PR China

The COVID-19 has disrupted health systems, even within the most developed economies, with case fatality rates in certain countries exceeding 10% [1].

COVID-19 deaths have been linked to diabetes and hypertension [2], which also correlate with obesity. As adipose tissue is prone to infection by the virus and produces high levels of inflammatory cytokines [3], it may feed the cytokine storm [4] suffered by many COVID-19 fatalities suggesting obesity could be the key factor.

COVID-19 nation death rates vary greatly, but countries with low obesity rates have consistently low fatality rates; specifically, Thailand, China, South Korea, Japan, and Pakistan are all countries of over 10 million population, but with a national obesity rate of 10% or less [5], which all have less than 50 deaths per million [1]. There are clearly a multitude of factors that contribute to the national death rates. However, countries with obesity rates of over 20% have far more variable mortality rates.

The poorest populations are most at risk of chronic conditions, which may put them at risk of dying from COVID19 [5], yet war torn countries such as Syria have not had the exponential increases in deaths observed in Europe and North America; suggesting that when comparing the effect on mortality, of different national strategies for COVID-19, national obesity rates should be considered.

National mitigation measures including lockdowns and social distancing may have to extend beyond 2020/21. During this time, individuals have been advised to do mild-to-moderate exercise as it can be of benefit in fighting viral diseases [6]. In addition, individuals should be advised to lose weight (with a mild caloric restriction) to minimise the risk of succumbing to COVID-19.

The pressing need for this is illustrated by the analysis of risk factors for critical illness; patients with a BMI>40 had a far higher risk (odds ratio OR 6.2) of requiring hospitalisation [7]. As over one-third of Americans, over one-fifth of Europeans and 13% of the adults in the world are obese [5], a very high percent of the population is at risk of severe symptomatology.

**Figure:**
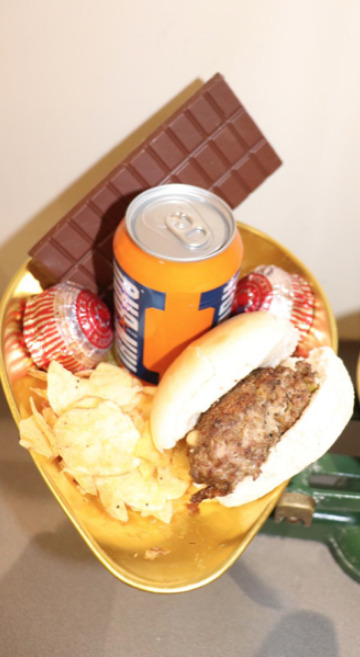
Photo: From Hamish Simpson’s own collection, used with permission.

Obesity rates rise with economic development of a country [8]. Paradoxically, the relationship of socioeconomic status (SES) to obesity changes, those of higher SES tend to be obese in lower income countries, whereas those of lower SES tend to be obese in high income countries. Thus, poorer individuals in wealthier nations are at particularly high risk of dying from COVID-19, yet due to inequalities, they will have most difficulty in accessing essential life care support. Unfortunately, interventions to prevent transmission have a particularly negative impact on these already impoverished communities. Ethnic minorities have been particularly hard hit by COVID-19, highlighting racial as well as economic inequalities [9].

With COVID-19, economies have been pulled into recession and unemployment rates have spiralled, exhausting national welfare safety nets and placing the burden onto the individual. Yet, those most at risk from this disease are the poorest in society, who have been most impacted by the mitigation strategies and those least able to access health care. Global health promotion strategies should emphasise the benefits of physical activity and weight loss interventions (such as those described by Brown et al [10]) in the fight against this pandemic, and country specific policies need to be developed quickly to support people who are most at risk.
